# Using an *in-vitro* biofilm model to assess the virulence potential of Bacterial Vaginosis or non-Bacterial Vaginosis *Gardnerella vaginalis* isolates

**DOI:** 10.1038/srep11640

**Published:** 2015-06-26

**Authors:** Joana Castro, Patrícia Alves, Cármen Sousa, Tatiana Cereija, Ângela França, Kimberly K. Jefferson, Nuno Cerca

**Affiliations:** 1 Centre of Biological Engineering, LIBRO – Laboratory of Research in Biofilms Rosário Oliveira, University of Minho, Campus de Gualtar, 4710-057, Braga, Portugal; 2 Instituto de Ciências Biomédicas Abel Salazar (ICBAS), University of Porto, Rua de Rua de Jorge Viterbo Ferreira 228, 4050-313 Porto, Portugal; 3 Department of Microbiology and Immunology, Virginia Commonwealth University, 1201 E Marshall St, Richmond, VA 23298-0678c, USA

## Abstract

*Gardnerella vaginalis* is the most common species found in bacterial vaginosis (BV). However, it is also present in a significant proportion of healthy women and *G. vaginalis* vaginal colonization does not always lead to BV. In an effort to better understand the differences between *G. vaginalis* isolated from women with a positive (BV) versus a negative (non-BV) diagnosis of BV, we compared the virulence potential of 7 BV and 7 non-BV *G. vaginalis* isolates and assessed the virulence factors related to biofilm formation, namely: initial adhesion and cytotoxic effect, biofilm accumulation, susceptibility to antibiotics, and transcript levels of the known vaginolysin, and sialidase genes. Furthermore, we also determined the ability of *G. vaginalis* to displace lactobacilli previously adhered to HeLa cells. Our results showed that non-BV strains were less virulent than BV strains, as suggested by the lower cytotoxicity and initial adhesion to Hela cells. Significant differences in expression of known virulence genes were also detected, further suggesting a higher virulence potential of the BV associated *G. vaginalis*. Importantly, we demonstrated that BV associated *G. vaginalis* were able to displace pre-coated vaginal protective lactobacilli and we hypothesize this to be a trigger for BV development.

Bacterial vaginosis (BV) is the most common vaginal disorder worldwide in women of reproductive age^1^. It is often characterized by the loss of normal vaginal flora, particularly *Lactobacillus* species, and overgrowth of anaerobes such as *Gardnerella vaginalis*^2^,^3^. While other bacterial species are also common, their role in BV is not clear and we recently demonstrated that *G. vaginalis* had significant higher virulence potential than other 29 BV associated species^4^. However, despite being the most prevalent and virulent species found in BV, *G. vaginalis* can also be a part of the vaginal microbiota in healthy women^5^,^6^. Consequently, there has been much debate in the literature concerning the contribution of *G. vaginalis* to the etiology of BV^4^,^7^. Reports indicate that *G. vaginalis* is a highly diverse taxon, both phenotypically and genotypically^8^,^9^. 8 biotypes of *G. vaginalis* have been identified by Piot^9^ based on the presence of β-galactosidase, lipase and hippurate hydrolysis activities, whereas Benito^8^ identified 17 biotypes not only based on the previous characteristics but also on fermentation of xylose, arabinose and galactose. Phenotypic diversity within *G. vaginalis* has also been described in terms of virulence factors, particularly production of sialidase^10^, cytotoxicity^7^ and ability to adhere and establish a biofilm on the vaginal epithelium^7^,^11^. Full genome sequencing of different *G. vaginalis* strains revealed significant differences between BV and non-BV isolates 7. This raised the question of whether there are distinct pathogenic and commensal lineages within this species. Thus, the present study aimed to isolate BV and non-BV associated *G. vaginalis* strains and to evaluate their virulence potential, using an *in vitro* biofilm model, by determining their ability to adhere to epithelial cells, to interfere with the displacement of healthy lactobacilli on epithelial cells, to grown as biofilm, to induce cytotoxic changes on epithelial cells, to express known virulence genes, and finally by determining their susceptibility to the antibiotics commonly used in BV treatment.

## Results

### Initial adhesion to human cervical HeLa cells and cytotoxic effect

**Figure 1:**
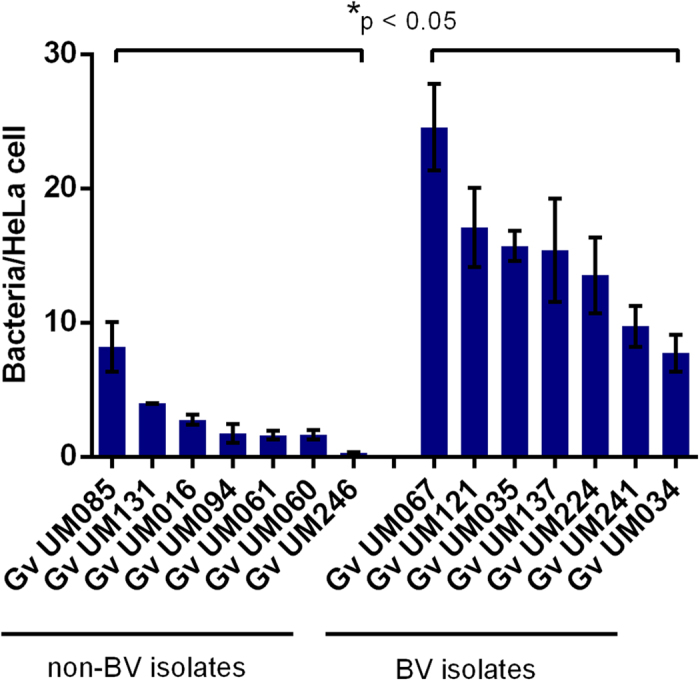
Initial adhesion of non-BV and BV *G*. *vaginalis* isolates to HeLa cells. Adhesion was microscopically quantified and expressed as the average ± SD number of bacteria per epithelial cell. ^*^Denotes significance differences between the 2 groups of *G. vaginalis* strains at same conditions (one-way ANOVA, p < 0.05).

**Figure 2:**
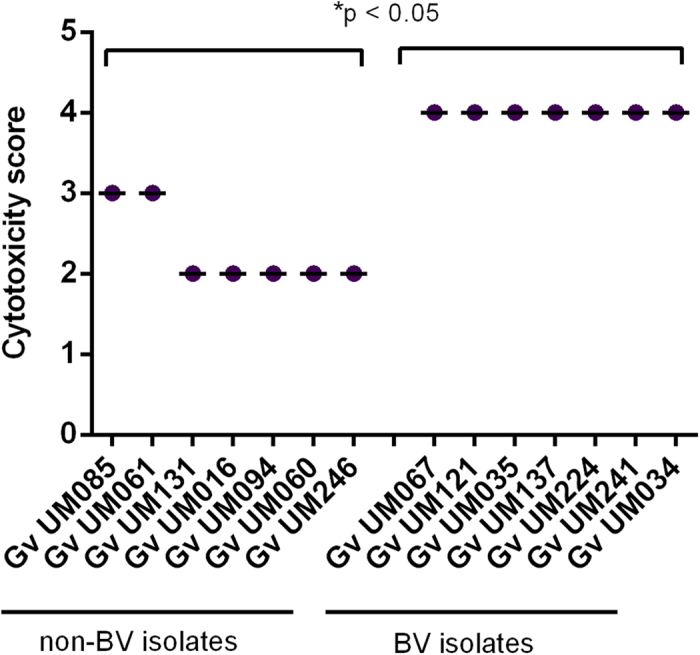
Cytotoxicity score of non-BV and BV *G*. *vaginalis* isolates. Cytotoxicity was scored as follows: 0, no difference between the experimental well and the control; 1, <25% cells were rounded; 2, 25–50% cells were rounded; 3, >50% cells were rounded; 4, >50% were rounded, with partial disruption of the monolayer; 5, complete disruption/absence of the monolayer. ^*^Values are significantly different between the 2 groups of *G. vaginalis* strains under the same conditions (one-way ANOVA, p < 0.05).

After isolating 7 BV and 7 non-BV associated strains of *G. vaginalis* (Supplementary Table S1) we first determined the ability of all strains to adhere to a monolayer of HeLa epithelial cells. As can be seen in Fig. 1, variations in adhesion were observed among the 14 isolates, with statistical differences between the 2 groups (p < 0.05). Importantly, BV isolates showed a greater ability to adhere to epithelial cells than non-BV isolates, with an average of 14.83 and 2.89 bacteria per HeLa cell, respectively. Cytotoxicity was also quantified in order to determine the capacity of the 2 groups of bacteria to induce cytotoxic changes in cell morphology on HeLa cells. Similar to the initial adhesion assays, BV isolates had a higher cytotoxicity score than non-BV isolates (p < 0.05; Fig. 2), which were only capable of causing slight morphological changes in HeLa monolayer.

### Biofilm formation

**Figure 3:**
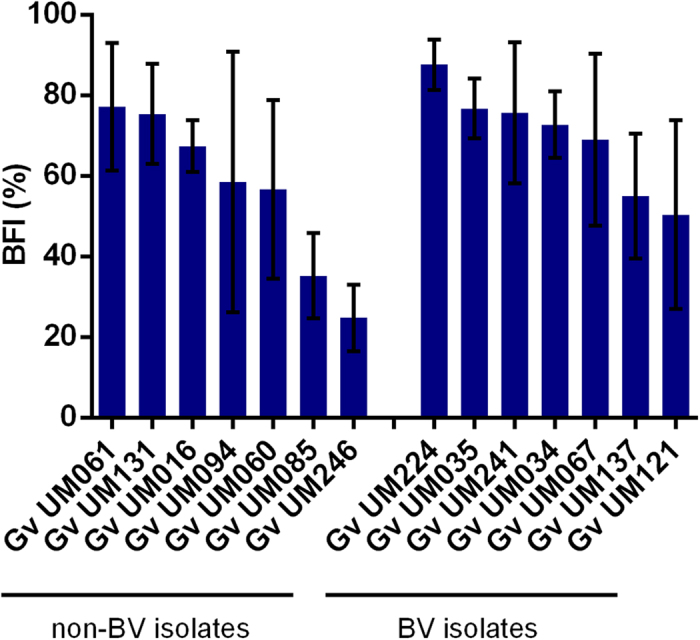
Intrinsic ability of non-BV and BV *G*. *vaginalis* isolates to form biofilms. The biofilm formation index (BFI) was defined as the average percentage of bacteria grown as biofilms, in the 3 media with higher biofilm growth for each *G. vaginalis* strains. The growth percentage as a biofilm for the 3 media was calculated using the equation OD_600nm_ biofilm/ (OD_600nm_ biofilm + OD_600nm_ planktonic) and represented as mean ± SD. No significant differences between non-BV and BV isolates were found to BFI (non-parametric Wilcoxon matched-pairs rank test, p = 0.176).

In order to determine the optimal medium for *in vitro* biofilm formation, all isolates were initially cultured anaerobically in 9 different media (Supplementary Table S2). As expected, *G. vaginalis* isolates formed different amounts of biofilm, depending on the growth media. To minimize the bias introduced by the growth media, we defined the biofilm formation index (BFI) as the average of growth in the 3 best growth media. Interestingly, our results showed that there were no significant differences in BFI between the 2 groups (p = 0.176), although there was a trend of higher BFI levels associated with the BV isolates (Fig. 3).

### Antimicrobial susceptibility

*In vitro* antimicrobial susceptibility of *G. vaginalis* was evaluated by determining the minimal inhibitory concentration (MIC) of metronidazole, tinidazole and clindamycin. Similar to the BFI determinations, no significant differences were found in antimicrobial resistance profiles between non-BV and BV isolates (p = 0.890; Table 1). Interestingly, all *G. vaginalis* strains tested exhibited intermediate resistance or resistance to metronidazole. Similarly, the strains exhibited intermediate resistance or resistance to tinidazole (86% of strains) while only 36% of strains were resistant to clindamycin.

### *G. vaginalis* ability to induce displacement of lactobacilli pre-adhered to epithelial cells

**Figure 4:**
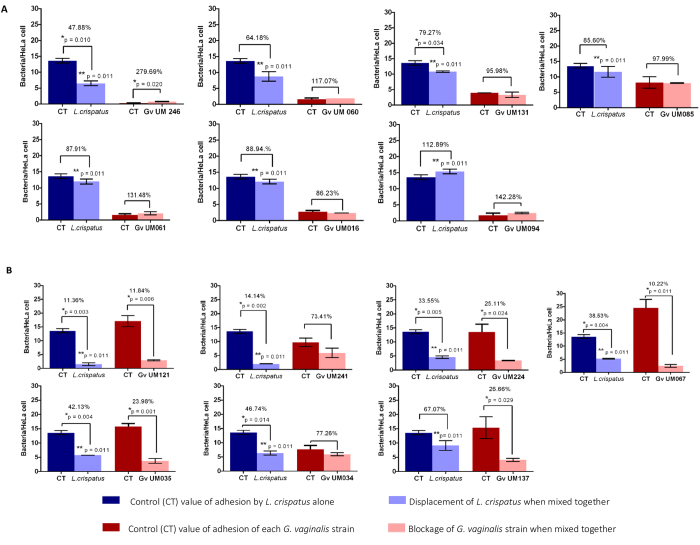
Influence of *L. crispatus* on *G*. *vaginalis* initial adhesion to HeLa cells. *L. crispatus* was pre-adhered to the epithelial cells. Subsequently, each *G. vaginalis* strain was added. (A) represents the non-BV isolates. (B) represents the BV isolates. Results are expressed as mean ± SD of bacteria/HeLa cell. The percentage indicated is the result of the variation in the final adhesion of *L. crispatus* and *G. vaginalis*, after *G. vaginalis* challenge to the pre-coated *L. crispatus*, as compared to the adhesion levels of each strain independently. ^*^Values are significantly different from the respective control (independent samples *t*-test, p < 0.05). ^**^Significant differences in the displacement of *L. crispatus* by two groups of *G. vaginalis* strains were found (one-way ANOVA, p = 0.011). No significant differences in the adherence of *G. vaginalis* were found between non-BV and BV isolates when mixed with *L. crispatus* (one-way ANOVA, p = 0.120).

We recently reported on the capacity of *G. vaginalis* to displace adherent vaginal lactobacilli from epithelial cells^12^. We sought to determine whether the non-BV and BV strains of *G. vaginalis* differed in their abilities to displace adherent lactobacilli populations. We found that, on average, BV isolates had a stronger ability to cause displacement of *L. crispatus* (63.78%) than non-BV isolates (19.05%, p *=* 0.011), as shown in Fig. 4. Also, similar to our previous observations^12^, *L. crispatus* inhibited the adherence of BV *G. vaginalis* isolates to the epithelial cells but failed to antagonize the adherence of non-BV isolates.

### Presence and expression of virulence genes

**Figure 5:**
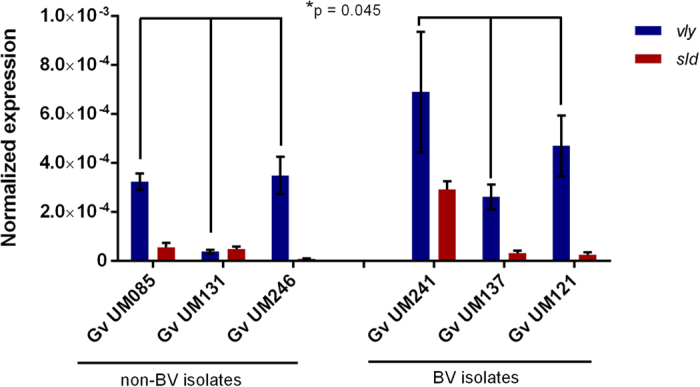
Expression of vaginolysin (*vly*) and sialidase (*sld*) by *G*. *vaginalis* isolates. Transcript levels within planktonic culture of the *G. vaginalis* strains were quantified. Results are expressed as normalized expression in relation to 16S rRNA and represented as mean ± SEM. ^*^Values are significantly different between non-BV and BV *G. vaginalis* strains to *vly* gene expression (one-way ANOVA, p = 0.045). No significant differences between two groups were found to *sld* gene expression (one-way ANOVA, p = 0.567).

To understand the role of virulence genes in non-BV and BV isolates of *G. vaginalis*, we initially determined whether the vaginolysin (*vly*) and sialidase (*sld)* genes were present in all 14 strains. As shown in table 2, no differences were found between the groups. Surprisingly, we verified that *vly* was absent in strains UM035 and UM224, as determined by PCR amplification with 2 independent pairs of primers, contrary to what was been described before^7^,^13^. Furthermore, this data was confirmed by amplifying and sequencing the flanking regions of *vly* (Supplementary Fig. S1). Since we did not find differences in the presence of these virulence genes between the 2 groups, we then analyzed the expression of those genes using a selection of 6 *G. vaginalis* strains (3 of each group) in which all strains carried the 3 genes of interest. Our data revealed differences in the expression of the tested genes (Fig. 5). Interestingly, the biggest difference found between the 2 groups was related to *vly* expression, in which BV isolates of *G. vaginalis* showed, on average, an expression 2-fold higher than non-BV isolates (p = 0.045). Nevertheless, no significant differences in expression of *sld* (p = 0.567) were detected between the 2 groups.

## Discussion

This study provides a more comprehensive understanding of the different *G. vaginalis* strains that can be found in the vaginal bacterial ecosystem, in health or disease. Clearly, all 7 strains isolated from women with BV were more virulent than the 7 non-BV strains. However, contrary to what was previously hypothesized, this increased virulence was not directly related to biofilm accumulation^7^, since all of our strains had similar biofilm formation, assessed in distinct growth media. On the other hand, the higher initial adhesion and cytotoxicity, as well as the ability to displace pre-adherent healthy vaginal lactobacilli, were important features of BV associated *G. vaginalis,* suggesting that the trigger for BV development could occur during the early stages of biofilm formation.

*G. vaginalis* is the most thoroughly studied BV associated microorganism but the fact that it is frequently present in healthy women casts doubt on its role in the etiology of BV^1^,^14^. Interestingly, it has been reported that certain biotypes of *G. vaginalis* are more frequently associated with BV^9^. However, functional microbiological studies addressing virulence properties of BV or non-BV strains are still scarce and often do not account for strain to strain variability^7^,^12^. We designed a series of *in vitro* experiments to compare the relative virulence capacities of BV and non-BV isolates of *G. vaginalis*. We used 7 different strains per group, to increase the confidence of the results.

We started by quantifying *G. vaginalis* initial adhesion to HeLa cells, since initial adhesion to the vaginal epithelium is a crucial step in BV development^15^ and the first step of biofilm formation^16^. Importantly, our data clearly showed that BV isolates adhered more avidly to the epithelial cells. Because the vagina is commonly colonized by *Lactobacillus* species^1^,^2^,^3^,^17^, we also explored the interaction between different *G. vaginalis* isolates and protective lactobacilli. The pathogenesis of BV is poorly understood and two different chains of events leading to BV have been proposed. One suggests that the population of lactobacilli is drastically reduced, by yet unknown factors, thus allowing the colonization by the multiple bacterial species associated with BV, while the other proposes that a single bacterial agent competes with lactobacilli, resulting in its overgrowth, later allowing other species to colonize the vaginal epithelium^3^. Recently, we showed that while one BV associated *G. vaginalis* strain was able to displace a protective layer of vaginal lactobacilli and colonize HeLa epithelium cells, this did not occur with a non-BV strain^12^. To confirm those findings, we analysed the ability of the *G. vaginalis* panel used in this study to displace *L. crispatus* previously adhered to the HeLa cells. Strengthening our previous observations, only BV associated strains of *G. vaginalis* were able to displace around 80% of the pre-coated lactobacilli (5 out of 7 strains). On the other hand, *L. crispatus* had a more pronounced effect in impeding the colonization by BV associated *G. vaginalis*. This data suggests that BV associated variants of *G. vaginalis* could be the primary pathogens in BV development, since this subset of strains have the ability to significantly displace vaginal lactobacilli, supporting one of the BV development models proposed^3^.

We also analyzed the ability of *G. vaginalis* to cause cytopathogenic changes in HeLa epithelial cells. We found that the BV isolates were significantly more cytotoxic, inducing rounding and lysis of HeLa epithelial cells, while non-BV *G. vaginalis* were unable to cause such cytopathogenic changes. The cytotoxicity activity of BV isolates could be due to a pore-forming toxin produced by *G. vaginalis*, vaginolysin, which is able to induce cell death and is thus a virulence factor^13^. Interestingly our data revealed that on average, BV isolates expressed 2-fold more *vly* than non-BV strains. However, strain to strain variability suggests that *vly* expression is not exclusive of BV associated *G. vaginalis*. Furthermore, sialidase could increase the cytotoxic activity of *G. vaginalis* and contribute to exfoliation and detachment of vaginal epithelial cells, by degrading mucins, which normally protect the epithelium^10^. Our studies did not reveal a direct relationship between sialidase expression and cytotoxicity, however, the epithelial monolayers used in our model do not produce mucins. Therefore, a different model system would be required to test this hypothesis.

It has also been described that as BV progresses, a highly structured polymicrobial biofilm develops on the vaginal epithelium and a major component of the biofilm is *G. vaginalis*^11^,^15^,^18^,^19^,^20^. Taking into consideration the differences in adhesion to epithelial cells, and the fact that initial adhesion does not always correlate to biofilm accumulation^21^, we characterized the intrinsic ability of *G. vaginalis* strains to grow as biofilms. Curiously, in our *in vitro* assay, BV isolates generally presented a higher BFI, however, differences in biofilm formation between the 2 groups did not reach statistical significance. Nevertheless, only 5 out of 7 non-BV isolates were able to grow preferentially as a biofilm (BFI >50%) while all 7 BV isolates analyzed showed a BFI >50%. Biofilm formation is an important virulence factor because it confers increased tolerance to antibiotics^22^ and antimicrobial byproducts produced by lactobacilli normally associated with the healthy vagina^18^. Importantly, we detected high levels of antimicrobial resistance in all isolates analyzed, confirming our previous reports^4^. Surprisingly, similar to the biofilm assay, no differences were detected between the two groups. Overall, *G. vaginalis* strains were more susceptible to clindamycin than to metronidazole or tinidazole, which was unexpected based on previous reports^23^,^24^.

This work clearly demonstrates strain differences between *G. vaginalis* isolates that could impact the ability of this organism to cause disease. However, the *in vitro* model of adherence used in this study is limited by the fact that cell monolayers of HeLa cells are not polarized, as are vaginal epithelial cells *in vivo*. The assay for biofilm formation was limited by the fact that the growth medium did not contain all of the factors found *in vivo*, and some *in vivo* cues may turn on expression of biofilm-related genes. Nevertheless, these limitations aside, *in vitro* models can be very informative, and are key to furthering our understanding of virulence potential of *G. vaginalis.*

Taking in consideration our novel findings and our previous observations^4^,^12^,^25^,^26^ we hypothesize that colonization by a subset of *G. vaginalis* is the trigger for BV development. By displacing lactobacilli, adhered *G. vaginalis* will then start to form a biofilm that will subsequently promote the incorporation of secondary colonizers and this mixed biofilm will ultimately become recalcitrant to antimicrobial therapy, similar to what has been described for oral biofilms^27^. Future genomic characterization of the non-BV and BV isolates of *G. vaginalis* will unveil the molecular mechanisms involved in these reported virulence differences. We envision that this will later impact novel diagnostic procedures and therapeutic options to treat BV.

## Methods

### Subject selection and sample collection

Vaginal samples were obtained from volunteers during private gynecology consult. All sampling was conducted in accordance with relevant guidelines and regulations and research approved by the University of Minho Institutional Review Board (approval number: SESVC 003-2013) in accordance with the Declaration of Helsinki and the guidelines of Good Clinical Practice. Written informed consent was obtained from all study participants prior to enrolment. Women were excluded from the study if they had any chronical disease. Classification of samples was done as before^28^. Briefly, BV diagnosis was first performed by the clinician, using the Amsel criteria^29^. Then based on the criteria for BV assessment developed by Nugent *et al.*^30^, participants with the Gram stain score of ≥7 were finally confirmed as BV (Supplementary Table S3). We also probed the samples with a novel PNA-FISH probe against *G. vaginalis*^31^.

### Bacterial isolation and identification

The presence of *G. vaginalis* in vaginal samples was further confirmed by PCR using an optimized protocol, as we previously described^32^. Samples positive for *G. vaginalis* were plated in columbia blood agar medium (Liofilchem, Roseto degli Abruzzi, Italy) with 5% (v/v) defibrinated horse blood (Oxoid Ltd., Basingstoke, Hants, United Kingdom) and incubated under anaerobic conditions, as described before^4^,^33^. Isolated bacteria were analyzed by Gram stain and subsequently identified by partial sequencing of 16S rRNA coding gene as described before^34^ (Eurofins, Germany). Nucleotide sequences obtained were compared to known sequences through BLAST software (NCBI, Bethesda, MD, USA). The primers used are listed in Supplementary Table S4. The accession number for these 14 strains are listed in Supplementary Table S1.

### Initial adhesion to epithelial cells and cytotoxicity assays

Initial adhesion to human cervical HeLa cells (ATCC CCL-2) and cytotoxicity assays were performed as described previously^4^. Briefly, for the adhesion assays, blind bacterial suspensions with a concentration of 1 × 10^8^ colony-forming units (cfu)/mL were added to a monolayer of HeLa cells for 30 minutes at 37 °C under anaerobic conditions. After washing the non-adherent bacteria, cells were fixed with methanol and adhesion was microscopically quantified as we previously described^4^. For the cytotoxicity assays, blind bacterial suspensions adjusted to 2.9 × 10^7^ cfu/mL were added to a monolayer of HeLa cells for 3 hours. Cytotoxicity was scored on a 0 to 5 scale^18^. Numeric scores were assigned as follows: 0, no difference between the test and the control; 1, 25% of the cells were rounded; 2, 25–50% of the cells were rounded; 3, 50% of the cells were rounded; 4, 50% cells were rounded, with partial disruption of the monolayer; and 5, complete disruption or absence of the monolayer. All experiments were performed in triplicate with technical replicates.

### Quantification of biofilm formation

Bacteria were grown in 9 different commercially available culture media, commonly used for biofilm growth: LB [composed by 10 g/L Tryptone (Liofilchem), 5 g/L yeast extract (Liofilchem) and 10 g/L of NaCl (Liofilchem)], MRS (Liofilchem), TSB (Liofilchem), sBHI [BHI (Liofilchem) supplemented with 2% (w/w) gelatin (Oxoid), 0.5% (w/w) yeast extract, 0.1% (w/w) starch (Thermo Fisher Scientific, Lenexa, KS, USA )], sBHIF [sBHI with 10% (v/v) FBS], and finally LBG, MRSG, TSBG and sBHIG supplemented with 0.25% (w/v) of glucose (Liofilchem)^4^. Biofilm formation assays were performed as described previously^7^,^18^. In brief, 200 μL of each bacterial suspension adjusted to 1 × 10^6^ cfu/mL was incubated in 96-well flat-bottom tissue culture plates (Orange Scientific, Braine L’Alleud, Belgium) at 37 °C for 48 hours under anaerobic conditions. Biofilms were first qualitatively evaluated with safranin staining^18^. Subsequently, the intrinsic ability of *G. vaginalis* strains to grow as biofilms was quantified, using the equation Optical Density (OD)_600nm_ biofilm / (OD_600nm_ biofilm + OD_600nm_ planktonic) as described by Harwich *et al.*^7^, for the 3 media that promoted the greatest biofilm growth. The biofilm formation index (BFI) was defined as the average biofilm quantity in the 3 selected growth media^4^. All assays were repeated 3 times with technical replicates.

### Antibiotic susceptibility

The susceptibility of *G. vaginalis* to antibiotics was evaluated by determining the minimal inhibitory concentration (MIC) of metronidazole, tinidazole and clindamycin. A pre-culture was first prepared for each isolate in sBHI by incubating at 37 °C under anaerobic conditions. After 24 hours, growth was confirmed by measuring the OD at 600 nm. MIC was determined by microdilution method in 96-well tissue culture plates^35^. All assays were repeated 3 times with technical replicates.

### *G. vaginalis* ability to induce displacement of lactobacilli pre-adhered to epithelial cells

The ability of *G. vaginalis* to displace *Lactobacillus crispatus* pre-adhered to epithelial cells was assessed using a protocol that we previously optimized^12^ with minor changes. Briefly, a suspension of 1.0 × 10^9^ cfu/mL of *L. crispatus* EX533959VC06 was added to each well of the 24-well plate containing the monolayer of HeLa cells. The plates were incubated for 4 hours at 37 °C in anaerobic conditions, at 0.081 *g (*PSU-10i, Biosan, Latvia). Subsequently, *G. vaginalis* strains (1.0 × 10^8^ cfu/mL) were added for 30 minutes under the same conditions as described above. Bacterial quantification was done as previously described^36^.

### PCR detection of virulence genes

Oligonucleotide primers for the detection of *vly* and *sld* genes were designed using the Primer3 software^37^ using the complete genome of *G. vaginalis* strain ATCC 14019 as a template. The 16S rRNA was used as internal control. Negative PCR results were confirmed using a second pair of independent primers. All primers used are listed in Supplementary Table S4. Genomic DNA was extracted as described before^32^ and the thermocycling program (Mini-MJ, Bio-Rad, Hercules, CA, USA) was performed using the DreamTaq PCR Master Mix 2x (Finnzymes, Espoo, Finland) and consisted on the following steps: 94 °C for 2 minutes followed by 40 cycles of 94 °C for 30 seconds, 58 °C for 30 seconds, 72 °C for 60 seconds and finally 72 °C for 5 minutes. The PCR product was then kept hold at 4 °C. PCR products were analyzed by gel electrophoresis with 1.5% agarose (Bioron, Ludwigshafen, Germany) and Orange G DNA loading dye (Thermo Fisher Scientific). All assays were repeated 3 times.

### Gene expression quantification

*G. vaginalis* strains were grown as described for the adhesion assays. Total RNA was extracted as previous described^38^. Briefly, genomic DNA was degraded with one step of DNase treatment (Fermentas, Lithuania) following manufacturer’s instructions. RNA concentration, purity and integrity was determined as described before^39^. Quantitative PCR (qPCR) was performed as previously described^38^ with some modifications. Briefly, qPCR was done using a CFX96^TM^ thermal cycler (Bio-Rad) with the following cycling parameters: 3 minutes at 95 °C, followed by 45 cycles of 10 seconds at 95 °C, 10 seconds at 58 °C and 15 seconds at 72 °C. The primer efficiency and the normalized gene expression was determined by using the delta Ct method (2^ΔCt^), a variation of the Livak method, where ΔC_t_ = C_t_ (reference gene) – C_t_ (target gene). All primer pairs had similar efficiencies. A control lacking the reverse transcriptase enzyme was included in each reaction. Gene expression assays were performed 3 independent times and in each time we had 3 qPCR wells per gene.

### Statistical analysis

The data were analyzed using the independent samples *t*-test, one-way analysis of variance (ANOVA), or non-parametric Wilcoxon matched-pairs rank test for the data that did not follow a normal distribution according Kolmogorov-Smirvon’s test, with the statistical software package SPSS 17.0 (SPSS Inc. Chicago, IL). The data were represented as mean ± standard deviation (SD) or as mean ± standard error of mean (SEM) at least 3 independent experiments. *P*-values of less than 0.05 were considered significant.

## Additional Information

**How to cite this article**: Castro, J. *et al.* Using an *in-vitro* biofilm model to assess the virulence potential of Bacterial Vaginosis or non-Bacterial Vaginosis *Gardnerella vaginalis* isolates. *Sci. Rep.* **5**, 11640; doi: 10.1038/srep11640 (2015).

## Supplementary Material

Supplementary Information

## Figures and Tables

**Table 1:** Minimum inhibitory concentration (MIC) of metronidazole, tinidazole and clindamycin for planktonic cells of *G. vaginalis* isolates.

**Strain**	**MIC range**		
	**Metronidazole**	**Tinidazole**	**Clindamycin**
**non-BV associated**			
*G. vaginalis* UM085	>[128]	[16]	<[0–01]
*G. vaginalis* UM061	[16]–[32]	[8]-[16]	<[0.01]
*G.vaginalis* UM131	>[128]	>[128]	>[128]
*G.vaginalis* UM016	[32]	[8]–[16]	<[0.01]
*G. vaginalis* UM094	>[128]	[32]	[0.5]
*G. vaginalis* UM060	>[128]	>[128]	<[0.01]
*G. vaginalis* UM246	[16]–[32]	[16]–[32]	>[128]
**BV associated**			
*G. vaginalis* UM067	[16]–[32]	[8]–[16]	<[0.01]
*G. vaginalis* UM121	[32]–[64]	[16]	>[128]
*G. vaginalis* UM035	[64]–[128]	[4]–[8]	<[0.01]
*G. vaginalis* UM137	[32]–[64]	[16]–[32]	>[128]
*G. vaginalis* UM224	[32]	[16]–[32]	>[128]
*G. vaginalis* UM241	[32]	[2]–[4]	<[0.01]
*G. vaginalis* UM034	>[128]	[32]–[64]	<[0.01]

Statistical analysis: no significant differences were found in antimicrobial resistance profiles between non-BV and BV associated isolates by the non-parametric Wilcoxon matched-pairs rank test (p = 0.890).

**Table 2:** Detection by PCR of the *vly* and *sld*genes in *G. vaginalis* isolates.

**Strain**	**Presence of virulence genes**		
	***vly***	***sld***	
**non-BV associated**			
*G. vaginalis* UM085	+	+	
*G. vaginalis* UM061	+	+	
*G. vaginalis* UM131	+	+	
*G. vaginalis* UM016	+	+	
*G. vaginalis* UM094	+	–	
*G. vaginalis* UM060	+	+	
*G. vaginalis* UM246	+	+	
**BV associated**			
*G. vaginalis* UM067	+	+	
*G. vaginalis* UM121	+	+	
*G. vaginalis* UM035	–	+	
*G. vaginalis* UM137	+	+	
*G. vaginalis* UM224	–	–	
*G. vaginalis* UM241	+	+	
*G. vaginalis* UM034	+	–	
