# Supplementary material for: Using an in-vitro biofilm model to assess the virulence potential of Bacterial Vaginosis or non-Bacterial Vaginosis Gardnerella vaginalis isolates
Source: Sci Rep. 2015 Jun 26;5:11640. doi: 10.1038/srep11640 (PMC4481526; doi:10.1038/srep11640)
Supplement: Supplementary Information [file srep11640-s1.doc]

**Using an *in-vitro* biofilm model to assess the virulence potential of Bacterial Vaginosis or non-Bacterial Vaginosis *Gardnerella vaginalis* isolates**

Joana Castro, Patrícia Alves, Cármen Sousa, Tatiana Cereija, Ângela França, Kimberly K. Jefferson & Nuno Cerca

**Supplementary data**

| Supplementary Table S1| Accession code of *G. vaginalis* strains | | | |
| --- | --- | --- | --- |
|  | **Strain** | **Accession number** |  |
|  | **non-BV associated** |  |  |
|  | *G. vaginalis* UM016 | KP996686 |  |
|  | *G. vaginalis* UM060 | KP996673 |  |
|  | *G. vaginalis* UM061 | KP996674 |  |
|  | *G. vaginalis* UM085 | KP996679 |  |
|  | *G. vaginalis* UM094 | KP996680 |  |
|  | *G. vaginalis* UM131 | KP996676 |  |
|  | *G. vaginalis* UM246 | KP996677 |  |
|  | **BV associated** |  |  |
|  | *G. vaginalis* UM034 | KP996684 |  |
|  | *G. vaginalis* UM035 | KP996685 |  |
|  | *G. vaginalis* UM067 | KP996675 |  |
|  | *G. vaginalis* UM121 | KP996681 |  |
|  | *G. vaginalis* UM137 | KP996682 |  |
|  | *G. vaginalis* UM224 | KP996678 |  |
|  | *G. vaginalis* UM241 | KP996683 |  |
| The partial *16S ribosomal RNA* gene sequence of *G. vaginalis* strains are deposited at NCBI. | | | |

| Supplementary Table S2| Qualitative analysis^a^ of biofilm formed by *G. vaginalis* strains in 9 different media | | | | | | | | | | | | |
| --- | --- | --- | --- | --- | --- | --- | --- | --- | --- | --- | --- | --- |
|  |  | **Media^b^** | | | | | | | | | |  |
|  | **Strain** | | **LB** | **LBG** | **MRS** | **MRSG** | **TSB** | **TSBG** | **sBHI** | **sBHIG** | **sBHIF** |  |
|  | **non-BV associated** | |  |  |  |  |  |  |  |  |  |  |
|  | *G. vaginalis* UM085 | | +- | +- | +- | +- | +- | +- | +- | +- | +- |  |
|  | *G. vaginalis* UM061 | | - | +- | - | - | +- | +- | ++ | +++ | +- |  |
|  | *G. vaginalis* UM131 | | +- | +- | +- | +- | +- | +- | +- | +- | +- |  |
|  | *G. vaginalis* UM016 | | +- | +- | - | - | +- | +- | ++ | ++ | ++ |  |
|  | *G. vaginalis* UM094 | | +- | +- | +- | +- | +- | +- | ++ | ++ | +- |  |
|  | *G. vaginalis* UM060 | | - | +- | - | - | - | - | +- | +- | +- |  |
|  | *G. vaginalis* UM246 | | +- | +- | +- | +- | +- | +- | +- | +- | +- |  |
|  | **BV associated** | |  |  |  |  |  |  |  |  |  |  |
|  | *G. vaginalis* UM067 | | - | - | - | - | - | - | +- | +++ | +- |  |
|  | *G. vaginalis* UM121 | | +- | +- | +- | +- | +- | +- | +- | ++ | +- |  |
|  | *G. vaginalis* UM035 | | - | - | - | - | +- | +- | ++ | ++ | ++ |  |
|  | *G. vaginalis* UM137 | | - | +- | - | - | +- | +- | ++ | +- | +- |  |
|  | *G. vaginalis* UM224 | | - | - | +- | +- | +- | +- | ++ | +++ | ++ |  |
|  | *G. vaginalis* UM241 | | +- | +- | +- | +- | +- | +- | ++ | ++ | ++ |  |
|  | *G. vaginalis* UM034 | | - | - | - | - | +- | +- | +- | +- | +- |  |
|  | ^a^ Biofilm formation was classified using the following scale: (-) no biofilm formed, (+-) formed medium biofilm, (++) good biofilm formation, (+++) strong biofilm formation in all tests.  ^b^ LB: luria broth, LBG: LB supplemented with 0.25% (w/v) glucose, MRS: de man-rogosa and sharpe agar, MRSG: MRS supplemented with 0.25% (w/v) glucose, TSB: tryptic soy broth, TSBG: TSB supplemented with 0.25% (w/v) glucose, sBHI: BHI supplemented brain heart infusion broth supplemented with 2% (w/w) gelatin, 0.5% (w/w) yeast extract, and 0.1% (w/w) starch, sBHIG: sBHI supplemented with 0.25% (w/v) glucose, sBHIF: sBHI supplemented with 10% (v/v) fetal bovine serum. | | | | | | | | | | |  |

| Supplementary Table S3| Characterization of vaginal samples | | | | |
| --- | --- | --- | --- | --- |
|  | **Vaginal Samples** | **Nugent score** | **Woman age (years)** |  |
|  | **BV negative** |  |  |  |
|  | UM016 | 0 | 45 |  |
|  | UM060 | 1 | 24 |  |
|  | UM061 | 3 | 38 |  |
|  | UM085 | 1 | 24 |  |
|  | UM094 | 2 | 30 |  |
|  | UM131 | 1 | 28 |  |
|  | UM246 | 4 | 19 |  |
|  | **BV positive** |  |  |  |
|  | UM034 | 7 | 66 |  |
|  | UM035 | 7 | 43 |  |
|  | UM067 | 9 | 54 |  |
|  | UM121 | 7 | 23 |  |
|  | UM137 | 9 | 22 |  |
|  | UM224 | 9 | 33 |  |
|  | UM241 | 8 | 20 |  |

|  | Supplementary Table S4| Primer sequences used for PCR and qPCR assays | | | | |  |
| --- | --- | --- | --- | --- | --- | --- |
|  | **Target** | **Primers sequence (5’to 3’)** | **T_melting_ (ºC)** | **Amplicon size (bp)** | **Reference** |  |
|  | *16s RNA* (Bacteria) | Fw AGA GTT TGA TCC TGG CTC AG | 55 | 789 | 34 |  |
|  | *16s RNA* (Bacteria) | Rv GGA CTA CCA GGG TAT CTA AT | 55 | 789 | 34 |  |
|  | *16s RNA (G*. *vaginalis)* | Fw CTC TTG GAA ACG GGT GGT AA | 62 | 300 | 32 |  |
|  | *16s RNA (G*. *vaginalis)* | Rv TG CTC CCA ATC AAA AGC GGT | 62 | 300 | 32 |  |
|  | Vaginolysin | Fw 1 CTCGCATGCAGTACGATTCT | 58 | 187 | This study |  |
|  | Vaginolysin | Rv1 TCTGGTGCATCAACGCTTAC | 58 | 187 | This study |  |
|  | Vaginolysin | Fw 2 gccagacagcttgaagaacc | 60 | 116 | This study |  |
|  | Vaginolysin | Rv 2 cagtgctcttgctggtggta | 60 | 116 | This study |  |
|  | Sialidase | Fw 1 CCGAATTTGCGATTTCTTCT | 54 | 189 | This study |  |
|  | Sialidase | Rv 1 CGTACGGAAGTTTTGGAAGC | 58 | 189 | This study |  |
|  | Sialidase | Fw 2 GGGTTTATGCACACGCTTTT | 56 | 131 | This study |  |
|  | Sialidase | Rv 2 GAAAATGCAGACAACGCAGA | 58 | 131 | This study |  |
|  | Reverse flanking region of vly | GGCGGAATTATGTGCGTTATTGG | 55 | 3334 | This study |  |
|  | Forward flanking region of vly | CATCTTCGCCAGCAACTTCC | 55 | 3334 | This study |  |


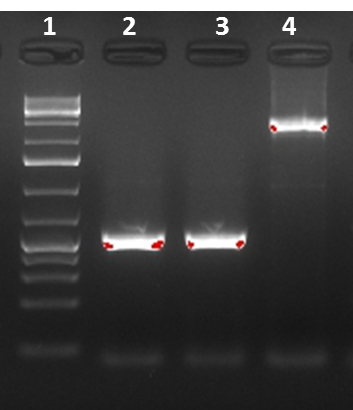


**Supplementary Figure S1 |** The absence of *vly* gene in *G. vaginalis* UM035 (2) and UM224 (3) strains was confirmed by PCR its flanking regions. *G. vaginalis* UM034 (4) was used as a positive control. 1Kb+ ladder was included (1). Subsequently the upper flanking regions were sequenced to confirm the identity of the genes. The sequencing results of the upper region are the following:

UM224: TAATTCGCCTTGCAAGCGACTGATTTCTTCGTCGCTAAGCGCAATCTGGGATAGAACGCCCAGATGCTCAATTTCTTCGCGTGTGAATGTTGGCATAACCTCAACTATATGTGTGATGCGTGACCTTTGCTATAGCAAAAATTATGCAAAAAGGGCTTTATCTTGTAGTCAAATCTTTTGAATCTACAGAGTAAAGCCCTTATTGTTTTTGCAAAATATTATTTGCAGAATATTTAAATATTTTAGATATCGCGATGCTTTTCAACAACGTGGCCAATTGCATACATGACTACGCCCCAAGCGAGAACCACCAAGCCTGATTGCCACCATGTGAAAATATATGCGTTTGGTGGAAGCTGTGCACCCGCATTAGGGGAGCCGCCCAAGAATTTCCCCACCGCTGTGGCTGGCAAAAGCTGTATAAGTATTGAATTCCACTTCGCGAAATTGCTTGCAAACATAATAATGCTAAGAACACTAGGCAAAATCACCACGGCTCCAATAACGCACATAAATTCCCGCCAAAAAA

UM035:TGACGTTTATTCGCCTTGCAAGCGACTGATTTCTTCGTCGCTAAGCGCAATCTGGGATAGAACGCCCAGATGCTCAATTTCTTCGCGTGTGAATGTTGGCATAACCTCAACTATATGTGTGATGCGTGACCTTTGCTATAGCAAAAATTATGCAAAAAGGGCTTTATCTTGTAGTCAAATCTTTTGAATCTACAGAGTAAAGCCCTTATTGTTTTTGCAAAATATTATTTGCAGAATATTTAAATATTTTAGATATCGCGATGCTTTTCAACAACGTGGCCAATTGCATACATGACTACGCCCCAAGCGAGAACCACCAAGCCTGATTGCCACCATGTGAAAATATATGCGTTTGGTGGAAGCTGTGCACCCGCATTAGGGGAGCCGCCCAAGAATTTCCCCACCGCTGTGGCTGGCAAAAGCTGTATAAGTATTGAATTCCACTTCGCGAAATTGCTTGCAAACATAATAATGCTAAGAACACTAGGCAAAATCACCACGGCTCCAATAACGCACATAATTCCGCCAAA
